# Impact of Improved Biomass and Liquid Petroleum Gas Stoves on Birth Outcomes in Rural Nepal: Results of 2 Randomized Trials

**DOI:** 10.9745/GHSP-D-20-00011

**Published:** 2020-09-30

**Authors:** Joanne Katz, James M. Tielsch, Subarna K. Khatry, Laxman Shrestha, Patrick Breysse, Scott L. Zeger, Naoko Kozuki, William Checkley, Steven C. LeClerq, Luke C. Mullany

**Affiliations:** a Department of International Health, Johns Hopkins Bloomberg School of Public Health, Baltimore, MD, USA.; b Department of Global Health, Milken Institute School of Public Health, George Washington University, Washington, DC, USA.; c Nepal Nutrition Intervention Project, Sarlahi, Kathmandu, Nepal.; d Institute of Medicine, Tribhuvan University, Kathmandu, Nepal.; e Department of Medicine, Johns Hopkins School of Medicine, Baltimore, MD, USA.

## Abstract

**Background:**

Few randomized trials have assessed the impact of reducing household air pollution from biomass stoves on adverse birth outcomes in low-income countries.

**Methods:**

Two sequential trials were conducted in rural low-lying Nepal. Trial 1 was a cluster-randomized step-wedge trial comparing traditional biomass stoves and improved biomass stoves vented with a chimney. Trial 2 was a parallel household-randomized trial comparing vented biomass stoves and liquid petroleum gas (LPG) stoves with a year’s supply of gas. Kitchen particulate matter of 2.5 μm or less (PM_2.5_) and carbon monoxide (CO) were assessed before and after stove installation. Prevalent and incident pregnancies were enrolled at baseline and throughout the trials. Birth anthropometry was compared across differing exposure times in pregnancy.

**Results:**

In trial 1, the mean 20-hour kitchen PM_2.5_ concentration was reduced from 1380 µg/m^3^ to 936 µg/m^3^. Among infants born before the intervention, mean birth weight and gestational age were 2627 g (SD=443) and 38.8 weeks (SD=3.1), and 39% were low birth weight (LBW), 22% preterm, and 55% small for gestational age (SGA). Adverse birth outcomes were not significantly different with increasing exposure to improved stoves during pregnancy. In trial 2, the mean 20-hour PM_2.5_ concentration was 885 µg/m^3^ in households with vented biomass and 442 µg/m^3^ in those with LPG stoves. Mean birth weight was 2780 g (SD=427) and 2742 g (SD=431), among households with vented and LPG stoves, respectively. Respective percentages for LBW, SGA, and preterm were 23%, 13%, and 42% in the vented stove group and not statistically different from 31%, 17%, and 42% in the LPG group.

**Conclusions:**

Improved biomass or LPG stoves did not reduce adverse birth outcomes. PM_2.5_ and CO following improved stove installation remained well above the World Health Organization indoor air standard of 25 µg/m^3^ or intermediate air quality guideline of 37.5 µg/m^3^. Trials that lower indoor air pollution further are needed.

## Abstract (precis)

Improved biomass stoves may not reduce indoor air pollution as much as is needed to have an impact on adverse birth outcomes.

## Teaser

**Key Findings:**

- Two trials in rural southern Nepal reduced indoor air pollution through improved biomass or liquid petroleum gas stoves, but levels were still much higher than World Health Organization standards.
- Exposure of pregnant women to these lower air pollution levels was not associated with reduced incidence of low birth weight, preterm birth, and small for gestational age.

**Key Implications:**

- Improved biomass stoves may not reduce indoor air pollution as much as is needed to have an impact on adverse birth outcomes.
- Emphasis should be placed on behavior to reduce use of biomass stoves when using improved ones for maximum reduction of pollution.

## INTRODUCTION

Low birth weight (LBW), comprising almost exclusively small for gestational age (SGA) and preterm births, is strongly associated with morbidity and mortality in infancy.^1^^,^^2^ Observational studies have shown associations between reported use of biomass fuel and these adverse birth outcomes but are subject to residual confounding even after adjustment for socioeconomic characteristics.^3^^–^^9^ One study showed an adjusted 43% increased risk of preterm birth and 21% increase in SGA with use of biomass fuel, but measures of exposure were limited to reported use of solid fuels.^3^ A meta-analysis of 19 studies found an 86.43 g reduction in birth weight and a 35% increase in LBW associated with biomass fuel exposure.^5^ Another meta-analysis of 5 studies showed that newborns in households using biomass fuels had a reduction in birth weight of 95.6 g and a 38% increase in LBW.^7^ Although multiple observational studies exist, only 2 randomized trials have examined the impact of reduced indoor air pollution on birth outcomes through the introduction of improved biomass or ethanol stoves.^10^^,^^11^ A trial in the highlands of Guatemala observed a 39% reduction in carbon monoxide (CO) levels, an increase in mean birth weight of 89 g, and a 26% lower rate of LBW among infants in households with improved biomass stoves compared with traditional ones.^10^ However, few pregnancies (n=266) were enrolled and impacts on birth outcomes were not statistically significant. A more recent trial from Ibadan, Nigeria, enrolled 324 pregnancies and found an 88 g higher mean birth weight and 40% reduced prevalence of preterm birth among infants born in households provided with ethanol stoves.^11^

In 2010, it was estimated that 2.8 billion people used solid biomass fuels.^12^ Hence, if reductions in indoor air pollution exposures can be shown to have an impact on adverse birth outcomes, such interventions could potentially lower morbidity and mortality rates for a large number of newborns worldwide.

If reduced indoor air pollution has an impact on adverse birth outcomes, interventions could potentially lower newborn morbidity and mortality rates worldwide.

We report here the results of 2 randomized trials to reduce indoor air pollution caused by open burning of biomass fuel sources in the home and the effects on mean birth weight and gestational age, as well as LBW, SGA, and preterm births.

## METHODS

### Population

Two sequential trials were conducted in the low-lying plains of southern Nepal (Sarlahi District) contiguous with Bihar, northern India. This area is rural and has almost universal use of traditional mud brick biomass stoves (wood, dung, crop waste). The population consists primarily of subsistence farmers with low literacy rates. About half of women deliver at home and the prevalence of LBW, preterm, and SGA births is high.^13^^,^^14^ In trial 1, the study area consisted of eligible households in 4 village development committees (VDCs) in Sarlahi. Eligible households in 2 of these 4 VDCs were enrolled in trial 2. Trial 1 was conducted between March 2010 and August 2012, and trial 2 between March 2013 and March 2014.

### Study Design

The study methods have been described in detail elsewhere.^15^ The aim of trial 1 was to examine whether lowering indoor air pollution by using an improved biomass stove could reduce acute lower respiratory illness in children and adverse birth outcomes among pregnant women. At the end of the first trial, indoor particulate matter (PM) was reduced by the improved stove but was still very high. Therefore, a second trial was designed to examine whether cleaner fuel, such as liquid petroleum gas (LPG), could further decrease the exposure and increase the likelihood of improved health outcomes.

For trial 2, because households from trial 1 and newly eligible households were enrolled and randomized, some pregnant women who were previously enrolled in trial 1 may have changed the type of stove they used, depending on the randomization in trial 2. A total of 126 women were still pregnant at the end of trial 1 and were eligible to enroll in trial 2.

**FIGURE 1.**
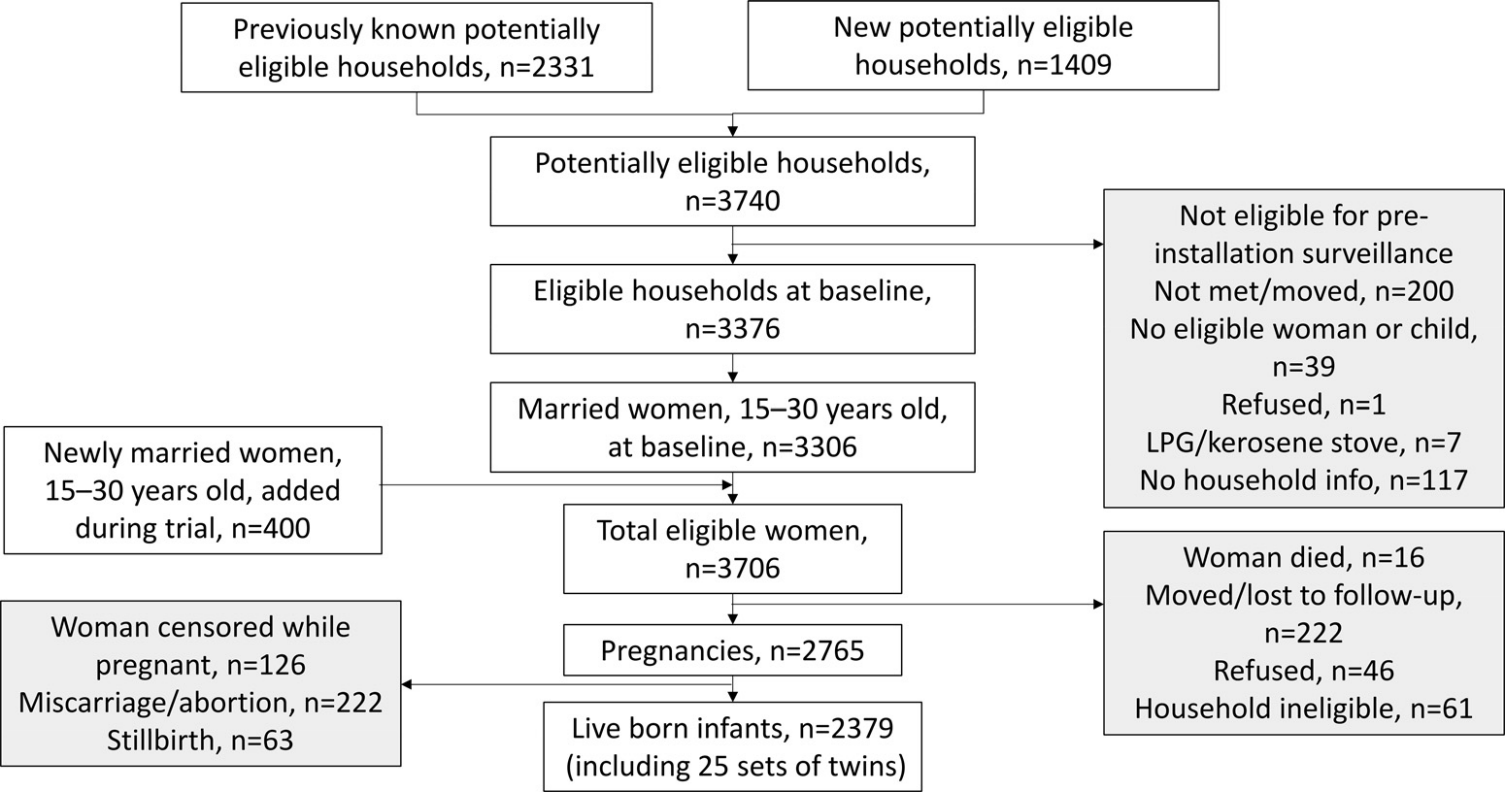
Nepal Cookstove Intervention Trial, Phase 1 CONSORT Diagram

Trial 1 was a randomized step-wedge community-based trial. Households were eligible if they did not have an LPG, electric, or improved vented biomass stove; had house materials that did not constitute a fire hazard with chimney installation; had at least 1 married woman 15–30 years of age or at least 1 child <36 months of age in the household at the start of the trial; and consented to participate. Women who were pregnant at the start of the trial were included and were assumed to have been exposed to traditional biomass stoves prior to enrollment in the trial. Over a 2-year period, these women were monitored for pregnancy with regular visits every 5 weeks. If women reported missing a period since the previous visit, project staff offered a pregnancy test; newly identified pregnant women were then followed until an outcome (miscarriage, live birth, or stillbirth). This process meant that for incident pregnancies, the recall time for date of last menstrual period was not very long, improving the quality of gestational age compared with asking about date of last menstrual period at delivery. The data collection for reproductive outcomes began with a 6-month trial of uniform surveillance in all enrolled households. This period was followed by a 12-month step-in period in which each household eventually had their open burning stove replaced with an improved version that had a chimney venting to the outside. The timing of the replacement from traditional biomass to improved biomass stove was randomized. Another 6-month period of surveillance for reproductive outcomes followed the 12 months of stove replacement. This design provided a similar time period and seasons before and after improved stove installation in the enrolled households (Figure 1).

**FIGURE 2.**
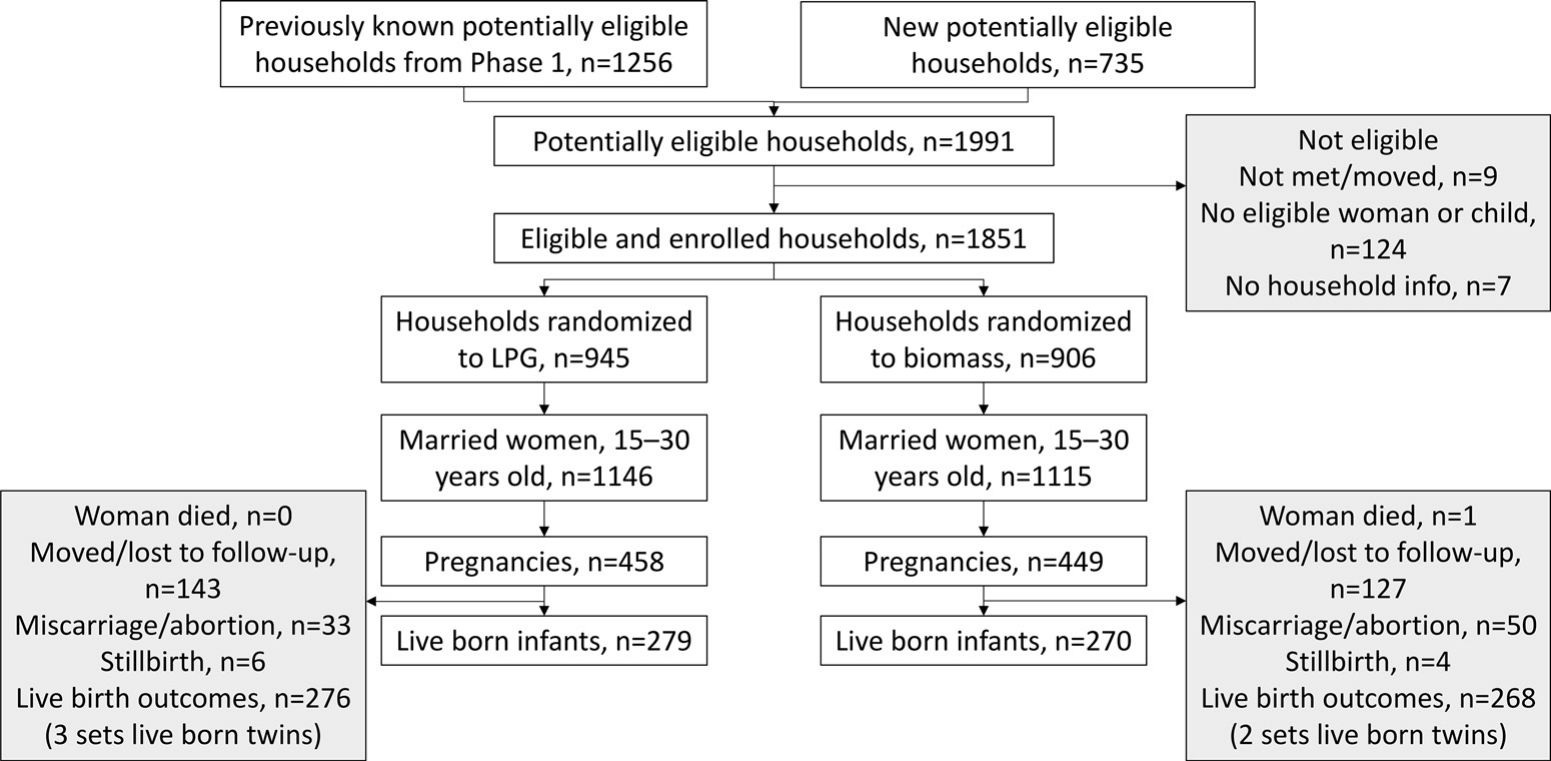
Nepal Cookstove Intervention Trial, Phase 2 CONSORT Diagram

In trial 2, households were eligible if they were in 2 of the 4 VDCs from trial 1, had at least 1 married woman 15–30 years of age or at least 1 child <24 months of age, and consented to participate. Study area households not enrolled in trial 1 were eligible for trial 2 if they met the same eligibility criteria as trial 1 (Figure 2). Households were individually randomized to use the improved biomass stove from trial 1 or to receive an LPG stove with a free year’s supply of gas. These households were followed for 1 year from the time of installation and birth outcomes within this time period were compared. Although this design was a traditional randomized trial, the length of time a pregnancy was exposed to the intervention varied.

### Randomization

In trial 1, 51 clusters of 20–30 households comprising about 25–40 children <3 years of age were identified. This number of households was the approximate number in which a single installation team could install improved stoves in 1 month. The clusters were then grouped such that all enrolled households could have stoves installed over 12 months, and those groups were randomized to the timing of receipt of the intervention. The numbers 1 through 12 were written on slips of paper, and senior project staff selected a number from a hat. The numbers represented the month of the year in which the intervention would be provided for each of the 12 groups of clusters. In trial 2, households were randomized stratified on VDC and ward (a smaller administrative unit, with 9 in each VDC) using the sample procedure in STATA.

### Sample Size

The sample size for both trials was based on the number of households needed to detect a difference in acute lower respiratory infection rates. The number of live births estimated to occur in trial 1 households was 2350 and 600 in trial 2. Given these live births, the detectable difference in birth weight was 57 g in trial 1 and 83 g in trial 2 with power of 80% and a type I error of 5%. Assuming 29% of infants would be born LBW (<2500 g) before the intervention (based on prior studies in this area), we had >80% power to detect a 20% relative reduction in LBW in trial 1, and 80% power to detect a 34% relative reduction in LBW in trial 2. Correlation of the outcomes within clusters was considered in the sample size calculations. Positive correlation increased the sample size required. The prevalence of adverse birth outcomes observed before the intervention in trial 1 was 39% LBW, 48% SGA, and 22% preterm. Trial 2 may have been underpowered because 9% fewer pregnancies were enrolled than the anticipated number of 600.

### Interventions

In trial 1, the intervention was a 2-burner biomass stove with a chimney to vent smoke to the outside. This stove was manufactured by Envirofit International (Colorado Springs, CO, USA), model G-3300 with G-3355 2 pot attachment.^15^ The stoves were installed by a specially trained study team, and household members were trained in their use and maintenance. Stove monitors visited households weekly to encourage and record stove use.

In trial 2, households were randomly assigned to either continue using the same Envirofit stoves as in trial 1 or were provided an LPG 2-burner stove with gas tank. Study workers provided each household with 1 tank per month, which was estimated to provide gas for an average household of 5 for the 12-month duration of the trial. At the end of the study, households were also provided an additional month of fuel. Households assigned to continued use of the Envirofit stove were provided LPG stoves and a 1-month supply of gas at the end of the trial.

### Exposure Measures

In trial 1, monitors to collect daily particulate matter of 2.5 μm or less (PM_2.5_), CO, humidity and temperature were deployed in each household at least once prior to and once post installation of the vented stove. On average, indoor air pollution was measured 6 months prior to and 6 months after stove installation. Measurements were made every 10 seconds over an average of a 21.7-hour period (from approximately 3:00 pm through 12:00 pm the next day). Lunch is not a usual meal in Nepal, so this monitoring period likely covered nearly all cooking events in a day. The package of instruments was placed approximately 1.5 m above the floor and about 1 m in front of the stove to best represent exposure of the person cooking. Details of the indoor air pollution monitoring and impact have been described elsewhere.^16^ We measured CO and PM_2.5_. We included CO measurements because we hypothesized that CO might be directly associated with adverse birth outcomes through the mechanism of oxygen displacement. In trial 2, the measurement taken post installation of the Envirofit stove in trial 1 was used as the PM_2.5_ exposure measure for trial 2 households randomized to retain the Envirofit stove. Exposure measures were obtained in LPG households post installation (between 1 and 12 months later, average 6 months) in trial 2 as a comparison. Temperature and humidity were measured with a HOBO U10 Temperature and Humidity (TH) Data Logger (Onset Computer Corporation, Pocasset, MA). CO was measured with the LASCAR CO data logger (EL-USB-CO300, Erie, PA). PM_2.5_ was measured using a DataRAM pDR 1000AN (Thermo Fisher Scientific Inc., Franklin, MA) as a nephelometer. These measures were then corrected for humidity based on calibration with gravimetric measures.^17^

### Outcome Measures

Pregnancies were followed to their outcomes. The family was asked to notify a local female study worker if the enrolled woman went into labor and a special team trained in infant anthropometry was dispatched to the household as soon after birth as possible to measure live born infants. Birth weights were measured with a Tanita BD-585 Pediatric Scale (Tanita, Tokyo, Japan) with an accuracy of 10 g. The scale was regularly calibrated prior to use. We used only weights obtained within 72 hours of birth. LBW was defined as <2500 g. Gestational age was estimated as weeks completed from date of last menstrual period (collected every 5th week by local female study workers visiting the homes of enrolled women) to birth. Gestational ages that fell outside a feasible range of 24 to <43 weeks were treated as missing. Preterm births were those occurring before 37 weeks completed gestation. SGA births were defined as those whose sex- and gestational-age-specific birth weights fell below the 10th percentile of the intergrowth population distribution using the upper bound of the weekly published data.^18^ Primary outcomes compared between stove interventions were mean birth weight, mean gestational age, LBW, preterm birth, and SGA.

### Statistical Analysis

For trial 1, descriptive demographic and socioeconomic characteristics are provided at baseline, prior to any stove installation. Given the step-wedge design, these characteristics were assumed not to change from pre to post installation. For trial 2, these same characteristics were compared between households that were randomized to the LPG intervention and those randomized to the improved biomass stoves to assess comparability between the 2 groups.

To characterize the impact of the vented biomass and LPG stoves, the mean daily PM_2.5_ in micrograms per cubic meter and CO in parts per million (ppm) were compared with traditional stove measures in both trials. In addition, we examined the number of hours the kitchen measures of PM_2.5_ were above 100 μg/m^3^ and above 1000 μg/m^3^ and CO measures were above 6 ppm and above 9 ppm and compared these measures of exposure between intervention groups. We chose these cut points for PM_2.5_ and for CO because we wanted to describe average daily exposure and an ambient level in the absence of a cooking event. The median daily exposure to PM_2.5_ was 1070 μg/m^3^ and the mean was 1380 μg/m^3^. Therefore, 1000 μg/m^3^ was chosen as a cut point.^16^ For CO, the median was 8.1 ppm and the mean was 11.0 ppm. We also used a statistical approach to determining when a cooking event was occurring (spikes in exposure could be seen). The lower cut point thus examined average ambient kitchen levels at times when cooking was not occurring. This cut point corresponded to PM_2.5_ and CO at about 100 μg/m^3^ and 6 ppm, respectively.

In trial 1, women could have experienced various lengths of exposure to the intervention stove while pregnant, ranging from no exposure to exposure for the entire pregnancy, given data collection occurred over 2 years. This exposure calculation applied to both existing (prevalent) and new (incident) pregnancies. Exposure for incident pregnancies was based on when in pregnancy the improved stove was installed. Given that the length of time exposed to the improved stove could affect the impact of the intervention, we stratified the effect of the intervention by time of exposure in utero (no exposure in pregnancy, >0 to <1/3, 1/3 to <2/3, 2/3 to <1, fully exposed). This was calculated as the time in pregnancy spent using each type of stove rather than trimesters exposed. Similarly, in trial 2, not all pregnancies were exposed to each randomized intervention for the entire length of pregnancy. However, given that trial 2 was a parallel individually randomized trial, we analyzed the birth outcomes comparing the 2 randomized groups without regard to length of exposure in utero because these were equal in both groups. In trial 2, 55% were enrolled in the first trimester, 29% in the second trimester and 16% in the third trimester.

Intervention effects in trial 1 were estimated using linear regression to compare the mean differences and 95% confidence intervals (CIs) in birth weight and gestational age between levels of exposure to the improved stove using no exposure as the reference. Secular trends in birth outcomes were adjusted for, using a cubic spline with knots every 100 days. We used this approach because there is strong seasonality in birth weights and to some extent in gestational age in this population.^13^ For LBW, preterm, and SGA, relative risks and 95% CIs were calculated with no exposure as the reference, using Poisson regression with robust variance estimation. In trial 2, the same birth outcomes were compared between the 2 treatment groups (Envirofit and LPG stoves) using linear regression for continuous outcomes and Poisson regression for binary outcomes.

In trial 1, the study design randomized the timing of intervention rather than geographic clusters. Groups that received the intervention each month during stove installation were deliberately scattered geographically across the study area to reduce within cluster correlation. Therefore, we did not adjust for cluster randomization. In trial 2, the unit of randomization was the household. During this 1-year period, 5 sets of twins were born. Hence we do not believe adjusting for multiple births in the same household would impact the outcomes.

Both trials were approved by the institutional review boards of the Johns Hopkins Bloomberg School of Public Health and the Institute of Medicine, Tribhuvan University, Kathmandu, Nepal. Verbal informed consent was obtained from all participating households and individuals and documented on data collection forms. The trials are registered at ClinicalTrials.gov (NCT 00786877).

## RESULTS

A total of 2,379 live born infants were enrolled in trial 1 and 270 and 279 in the Envirofit and LPG stove groups in trial 2, respectively (Figures 1 and 2). Neither maternal nor infant characteristics differed between the randomized arms in trial 2 (Table 1). The only notable difference between trials 1 and 2 was the percentage of deliveries at a facility, which was due to a strong secular trend in facility deliveries over the study time period. This trend was likely a result of a conditional cash transfer program implemented by the government of Nepal to incentivize women to deliver at facilities during this time.

**TABLE 1.** Baseline Characteristics of Mothers and Infants in 2 Randomized Trials of Improved Cookstoves in Rural Nepal

	**Trial 1 (N=2379)**		**Trial 2**			
			**Vented Biomass (N=270)**		**LPG (N=279)**	
	**No. (%)**	**(%)**	**No.**		**No.**	
Sex of newborn						
Male	1216	51.1	149	55.2	149	53.4
Female	1163	48.9	121	44.8	130	46.6
Maternal age at delivery,^a^ mean (SD), y	2379	23.5 (4.0)	270	24.5 (3.7)	279	23.0 (3.6)
<18	157	6.6	15	5.6	17	6.1
18–35	2219	93.3	255	94.4	262	93.9
≥35	3	0.1	0	0	0	0
Parity, mean (SD)	2374	1.7 (1.6)	266	2.8 (1.7)	275	1.6 (1.3)
0	578	24.4	40	15.0	53	19.3
1–3	1524	64.2	199	74.8	190	69.1
≥4	272	11.5	27	10.2	32	11.6
Maternal height, mean (SD), cm	2341	150.1 (5.6)	245	149.4 (5.4)	250	150.3 (5.2)
<145	357	15.3	42	17.1	29	11.6
145–149	654	27.9	81	33.1	83	33.2
150–154	800	34.2	80	32.7	91	36.4
≥155	530	22.6	42	17.1	47	18.8
Location of delivery	2353		256		266	
Facility	483	21.4	161	62.9	169	63.5
Home or outdoors	1807	78.6	95	37.1	97	36.5
Maternal education, mean (SD), y	2370	1.5 (3.2)	270	1.4 (3.2)	278	1.9 (3.6)
0	1827	77.1	214	79.3	209	75.2
1–9	398	16.8	41	15.2	42	15.1
≥10	145	6.1	15	5.6	27	9.7

Abbreviations: LPG, liquid petroleum gas; SD, standard deviation.

a Some maternal ages at delivery were greater than the eligibility upper limit of 30 years at the time of enrollment.

In trial 1, the mean kitchen-based 1-day average PM_2.5_ measurements in households using traditional stoves was 1380 (95% CI=1336, 1425) μg/m^3^ (Table 2). The improved biomass stove reduced PM_2.5_ to 936 (95% CI=895, 978) μg/m^3^. In trial 2, the improved biomass stove had a similar PM_2.5_ concentration to those in trial 1 at 885 (95% CI=810, 959) μg/m^3^, while the LPG stoves had a lower mean concentration of 442 (95% CI=405, 482) μg/m^3^. A small study done after the end of both trials to compare ambient kitchen to personal exposure in women of childbearing age showed that kitchen exposure overestimated personal exposure for traditional and improved biomass stoves, but was similar for LPG exposure (data not shown).

**TABLE 2.** CO and PM_2.5_ Kitchen-Based Concentrations in Households in 2 Randomized Trials Attributable to Improved Stoves, Adjusted for Potential Confounding, Rural Nepal

	**Trial 1**		**Trial 2**	
**Measures of Exposure**	**Traditional Biomass** **N=2963**	**Vented Biomass** **N=2752**	**Vented Biomass** **N=659**	**LPG** **N=661**
Total daily PM_2.5_, mean (95% CI), μg/m^3^	1380 (1336, 1425)	936 (895, 978)	885 (810, 959)	442 (405, 482)
Time above 100 μg/m^3^, mean (95% CI), hours, % sampling time	14.4 (14.2, 14.6), 66	11.7 (11.6, 11.8), 54	10.3 (10.0, 10.7), 47	9.2 (0.8, 9.6), 42
Time above 1000 μg/m^3^, mean (95% CI), hours, % sampling time	5.3 (5.2, 5.4), 24	3.9 (3.8, 4.0), 18	3.6 (3.3, 3.8), 17	2.3 (2.1, 2.4), 11
	n=2011	n=1848	n=526	n=522
Total daily CO, mean (95% CI), ppm	11.0 (10.6, 11.4)	6.7 (6.4, 7.1)	5.5 (5.0, 6.0)	1.7 (1.5, 1.9)
Time above 6 ppm, mean (95% CI), hours, % sampling time	6.6 (6.4, 6.8), 30	4.3 (4.1, 4.4), 20	3.4 (3.1, 3.6), 16	1.1 (1.0, 1.3), 5
Time above 9 ppm, mean (95% CI), hours, % of sampling time	4.9 (4.7, 5.0), 23	3.2 (3.1, 3.4), 15	2.6 (2.4, 2.9), 12	0.7 (0.6, 0.8), 3

Abbreviations: CI, confidence interval; CO, carbon monoxide; LPG, liquid petroleum gas; PM_2.5_, particulate matter of 2.5 μm or less.

a Adjusted for rainfall and temperature on the day of measurement.

Adherence to the intervention was measured at weekly visits in both trials. In trial 1, at 90% of weekly visits, households reported using a stove other than the intervention stove (Envirofit) following its installation. In trial 2, study workers provided a standard canister of gas monthly. There were no gaps in delivery. In 11 of 53,007 weekly adherence visits in the LPG group, the household reported being out of gas. In 96 visits, the household reported that the gas regulator was damaged or not working. In trial 2, at 53% of weekly visits, households reported using a stove other than the LPG stove.

Trial 1 provided no evidence that any of the reproductive outcomes were affected by installation of the improved biomass stove (Table 3). Differences or relative risks were not statistically different from 0 or 1, respectively, and there was no dose response effect according to level of exposure. Even comparisons between no exposure in pregnancy versus exposure for the entire pregnancy did not indicate any effect on any birth outcomes. In trial 2, there was no statistically significant difference in mean birth weight, prevalence of LBW, preterm birth, or SGA among those pregnancies in the LPG households compared with Envirofit households (Table 4).

**TABLE 3.** Birth Outcomes, Adjusted^a^ Mean Differences, and Adjusted Relative Risks by Maternal Exposure Time to Improved Stove During Pregnancy, Trial 1, Rural Nepal, N=2379

	**Time During Pregnancy Mother Exposed to New Stove, %**				
	**0**	**<33**	**33–65**	**66–99**	**100**
Number of infants gestational age recorded	943	165	141	125	474
Gestational age, mean (SD),^b^ week	38.6 (2.7)	38.4 (3.1)	39.2 (2.0)	38.8 (2.7)	38.5 (2.7)
Gestational age,^b^ mean difference (95% CI), week	Reference	−0.51 (−1.03, 0.01)	0.27 (−0.30, 0.85)	−0.24 (−0.87, 0.39)	−0.75 (−1.36, −0.14)
Preterm, %	22.5	23.6	13.5	21.6	22.2
Preterm,^b^ RR (95% CI)	1.00	1.38 (0.97, 1.97)	0.81 (0.50, 1.32)	1.41 (0.91, 2.20)	1.66 (1.08, 2.57)
Number of infants with birth weight measured	588	133	116	104	360
Birth weight, mean (SD),^c^ g	2630 (443)	2628 (443)	2647 (418)	2676 (408)	2657 (439)
Birth weight,^c^ mean difference (95% CI), g	Reference	−12.8 (−107.1, 81.4)	−7.7 (−112.7, 97.4)	28.9 (−87.2, 145.0)	−5.5 (−122.6, 111.6)
Low birth weight, %	38.6	39.9	30.2	33.7	32.2
Low birth weight,^c^ RR (95% CI)	1.00	1.08 (0.82, 1.41)	0.83 (0.59, 1.17)	0.92 (0.63, 1.34)	0.92 (0.65, 1.30)
Number of infants with both gestational age and birth weight recorded	522	118	102	93	331
Gestational age, mean (SD),^d^ week	38.7 (2.6)	38.6 (25.6)	39.2 (2.0)	38.9 (2.8)	38.4 (2.7)
Gestational age,^d^ mean difference (95% CI), week	Reference	−0.39 (−1.01, 0.22)	0.16 (−0.53, 0.84)	−0.28 (−1.03, 0.47)	−0.97 (−1.73, −0.20)
Preterm,^d^ %	20.7	22.0	12.8	20.4	23.3
Preterm,^d^ RR (95% CI)	1.00	1.41 (0.91, 2.18)	0.84 (0.46, 1.53)	1.51 (0.86, 2.62)	2.01 (1.13, 3.56)
Small for gestational age, %	47.5	52.5	55.9	50.5	44.1
Small for gestational age,^d^, RR (95% CI)	1.00	1.14 (0.90, 1.44)	1.21 (0.95, 1.54)	1.11 (0.83, 1.48)	1.00 (0.74, 1.34)

Abbreviations: CI, confidence interval; RR, relative risk; SD, standard deviation.

a Adjusted for secular trend (cubic spline every 100 days) and sex of infant.

b Gestational age includes any measures 24 to <43 weeks (5.8% of gestational ages fell outside this range).

c Birth weight includes all measured within 72 hours.

d Gestational age and preterm among those with birth weights within 72 hours of birth.

**TABLE 4.** Birth Outcomes by Randomization to Improved Stove Type, Trial 2, Rural Nepal

	**Stove Type**	
	**Vented**	**LPG**
Number of infants gestational age recorded	248	243
Gestational age, mean (SD),^a^ week	39.2 (2.2)	39.0 (2.4)
Difference (95% CI)	−0.3 (−0.7, 0.2)	
Preterm,^a^ %	13.3	19.4
RR (95% CI)	1.45 (0.97, 2.19)	
Number of infants with birth weight measured	188	207
Birth weight, mean (SD),^b^ g	2780 (427)	2742 (431)
Difference (95% CI)	−37 (−122, 47)	
Low Birth Weight,^b^ %	23.4	31.4
RR (95% CI)	1.34 (0.97, 1.86)	
Number of infants with both gestational age and birth weight recorded	176	184
Gestational age, mean (SD),^c^ week	39.2 (2.4)	39.0 (2.3)
Difference (95% CI)	−0.2 (−0.7, 0.3)	
Preterm,^c^ %	15.3	19.6
RR (95% CI)	1.28 (0.81, 2.01)	
Small for Gestational Age,^c^ %	47.7	46.7
RR (95% CI)	0.98 (0.79, 1.21)	

Abbreviations: LPG, liquid petroleum gas; RR, relative risk; SD, standard deviation.

a Gestational age between 24 and <43 completed weeks (3.5% of gestational ages fell outside this range).

b Birth weights taken within first 72 hours of birth.

c Gestational age and preterm among those with birth weights within 72 hours of birth.

## DISCUSSION

Using a step-wedge design, we were unable to show an impact of an improved biomass stove on any of our measured birth outcomes. No differences were seen in a comparison of no exposure and full exposure to the intervention in pregnancy. The number of infants in each group was sizeable (588 [no exposure] versus 406 [full exposure] for birth weight within 72 hours, and 955 versus 574 for gestational age). Similarly, we were unable to show a difference between improved biomass and LPG stoves in a parallel randomized trial. For households with LPG stoves, the number of participants with exposure for their entire pregnancy and those with no exposure for their entire pregnancy was too small for any meaningful comparison in trial 2, but the sample size for the standard intent to treat was adequate to detect a meaningful difference.

We were unable to show an impact of an improved biomass stove on any of our measured birth outcomes.

In trial 1, the same women crossed over from one intervention to another and would therefore have had the same underlying risks and morbidities in the same pregnancy; hence we did not adjust for morbidity or other characteristics. Trial 2 was randomized to one intervention or the other. Table 1 shows comparability of demographic and maternal characteristics, and randomization would make it likely that other morbidities were comparable as well.

Meta-analyses of observational studies estimate about 30% to 80% increased risk of LBW and 80–100 g lower mean birth weight with high exposure to indoor air pollution based on a mix of self-reported fuel use and measurements of PM_2.5_ and CO.^7^^–^^9^^,^^19^ The randomized trial (Guatemala) found a 26% reduction in LBW after adjustment, but the sample size was small (a total of 179 infants weighed within 48 hours) and the authors used a per protocol analysis to account for variability in several factors.^10^ The trial in Nigeria found an 88 g higher mean birth weight in those exposed to clean versus biomass fuel.^11^ This difference was statistically significant, but the researchers did not report the impact of the intervention on LBW. Preterm prevalence was also lower in the clean fuel group, but the numbers were small (10 versus 16 cases). The indoor air pollution levels in Nigeria were much lower than those measured in either of the trials in Nepal. Our kitchen CO measures were higher for both traditional and improved biomass stoves than in Guatemala, but the measures in that study were personal rather than ambient. Hence it is difficult to compare exposures between Nepal and Guatemala. When comparing no exposure with full exposure to improved biomass stoves in trial 1, we found a 16% reduction in LBW and a 23 g increase in mean birth weight, neither of which were statistically different, and no dose response to exposure in utero.

Although some evidence exists for an effect of improved biomass stoves and theoretically clean fuel stoves such as LPG, the levels of exposure to the traditional stoves were exceedingly high in our study population relative to other studies. The improved biomass stove reduced ambient kitchen PM_2.5_ and CO significantly, but the mean PM_2.5_ was still more than 37 times higher than the World Health Organization (WHO) standard. LPG stoves reduced PM_2.5_ exposure significantly from that of improved biomass stoves, but these levels were still 18 times higher than the WHO standard of 25 μg/m^3^ or lower. Reasons for continued high exposures despite the use of clean fuels likely included concurrent use of an open burning biomass stove within the household, high levels of dust in the air, and contamination from adjacent households where traditional open-burning continued to be practiced. These results indicate the need to further study stove design and stove use behaviors, as well as how these behaviors influence the use of these stoves. Although we eliminated cost barriers to the use of LPG stoves (by providing both stove and gas supply), such barriers are real and would likely further compromise the effectiveness of this intervention.

The improved biomass stove reduced ambient kitchen PM and CO significantly, but the mean PM was still more than 37 times higher than the WHO standard.

The strengths of these trials include randomization to timing of intervention (trial 1) and standard randomization (trial 2). These 2 sequential trials provided different levels of exposure by which to assess health impacts, providing an ability to assess a dose response to the exposure.

### Limitations

These trials have several limitations. First, gestational age was not measured using ultrasound but rather the date of the last menstrual period. This method could lead to some misclassification of preterm birth. A primary limitation of these trials was that the exposures, while lower than those due to the traditional stoves, were still very high with the improved biomass stoves. Although the LPG stoves further lowered exposure, these remained high. Use of a stove other than the intervention stove occurred at least once per week in about 90% of households in trial 1 and 50% in trial 2. Also, we did not measure ambient outdoor exposures, which could have contributed to the high exposures seen in both groups. Furthermore, many pregnancies were only partially exposed to the lower levels of PM_2.5_ and CO because the improved biomass or LPG stove was installed part way through the pregnancy. Sixty-seven percent of infants were weighed within24 hours, 72% within 48 hours, and 75% within 72 hours. Because we did not measure weights at the time of birth, and because infants lose weight within a few days of birth, our estimates of LBW are likely overestimates of the true LBW. However, because of randomization, this bias should be similar in both groups. These constraints may explain the lack of effect on birth outcomes. Another limitation is that exposures were ambient kitchen rather than personal measures, and the kitchen measures were likely an overestimate of the personal exposures.

## CONCLUSION

In summary, these analyses do not provide any evidence that the introduction of an improved biomass stove reduced adverse birth outcomes compared with open-burning biomass or LPG stoves. The reduction in PM_2.5_ and CO achieved by improved stoves was possibly inadequate to demonstrate any protective effect on this population. Despite clean fuel reducing indoor PM_2.5_ further, exposure levels were still very high, likely due to stove stacking and possibly high levels of ambient outdoor pollution, and may explain the lack of effect with this intervention. Further work is needed to assess the impact of lower exposures to PM_2.5_ and CO for the entire length of pregnancy on adverse birth outcomes.

## Notes

**Peer Reviewed**

**First published online:** July 17, 2020
